# Influence of Herbal Complexes Containing Licorice on Potassium Levels: A Retrospective Study

**DOI:** 10.1155/2014/970385

**Published:** 2014-06-19

**Authors:** WooSang Jung, SeungWon Kwon, JinWook Im, SeongUk Park, SangKwan Moon, JungMi Park, ChangNam Ko, KiHo Cho

**Affiliations:** ^1^Department of Cardiovascular and Neurologic Diseases, Kyung Hee University Oriental Medicine Hospital, 1 Hoegi-dong, Dongdaemun-gu, Seoul 130-702, Republic of Korea; ^2^Department of Cardiovascular and Neurologic Diseases, Kyung Hee University Hospital at Gangdong, Seoul 134-727, Republic of Korea

## Abstract

To observe the influence of these complexes on potassium levels in a clinical setting, we investigated the influence of herbal complexes containing licorice on potassium levels. We retrospectively examined the medical records of patients treated with herbal complexes containing licorice from January 1, 2010, to December 31, 2010. We recorded the changes in the levels of potassium, creatinine, and blood urea nitrogen and examined the differences between before and after herbal complexes intake using a paired* t*-test. In addition, we investigated the prevalence of hypokalemia among these patients and reviewed such patients. We identified 360 patients who did not show significant changes in the levels of potassium and creatinine (*P* = 0.815, 0.289). We observed hypokalemia in 6 patients. However, in 5 patients, the hypokalemia did not appear to be related to the licorice. Thus, we could suggest that herbal complexes containing licorice do not significantly influence the potassium levels in routine clinical herbal therapies. However, we propose that follow-up examination for potassium levels is required to prevent any unpredictable side effects of administration of licorice in routine herbal medicine care.

## 1. Introduction

Licorice has been used as a medicinal agent for patients of all ages and in East Asian countries. Licorice has been used for the treatment of peptic ulcer, as an anti-inflammatory agent, expectorant, drink, candy, and sweetener [1, 2]. In particular, in Korean medicine, licorice has been used in many herbal prescriptions for the treatment of sore throat, cough, and wounds and for neutralizing the poisonous effects of other herbs such as* Aconiti Ciliare Tuber*,* Ephedrae Herba,* or* Rhei Rhizoma*. Therefore, East Asian people who prefer taking herbal complexes for treating their various symptoms frequently consume licorice.

Licorice was reported to cause hypokalemia for the first time in 1950 [3]. Subsequently, many studies have shown that licorice can induce hypokalemia, muscle weakness, and hypertension [1, 4–17]. In serious situation, licorice consumptions can cause rhabdomyolysis with generalized muscle aches or weakness, hematuria, hypokalemia, and renal dysfunction [8–10]. These studies suggest that long-term use and overdose of licorice can lead to severe hypokalemia and can be life threatening. However, previous studies only included case reports.

In the present study, we investigated the influence of herbal complexes containing licorice on potassium levels in 360 cases. We aimed to observe the influence of such herbal complexes on potassium levels in a clinical situation.

## 2. Methods

We examined the medical records of patients who were treated with herbal complexes containing licorice from January 1, 2010, to December 31, 2010, at the Kyung Hee University Oriental Medicine Hospital, Seoul, Korea. From these patients, we selected subjects whose potassium levels were stable before the administration of herbal medication and whose potassium levels were in the normal range. Thus, we excluded patients with hypokalemia or hyperkalemia at the starting point of the study. Further, we selected subjects whose herbal medication and existing medications such as antiplatelet agents, antihypertensive agents, antidiabetic agents, statins, or nonsteroidal anti-inflammatory drugs had not changed during followup. If the composition of the herbal complexes or the dose of licorice or other medications changed, we assumed that followup was completed. We recorded the patients' characteristics, reasons for using herbal complexes, dose of licorice in each patient, duration of herbal medication intake, and changes in the levels of potassium, blood urea nitrogen (BUN), and creatinine. To measure the differences in potassium levels during administration of herbal medicines containing licorice (before and after herbal medicine intake), we used a paired* t*-test. Statistical analysis was performed using SPSS for Windows, version 10.0 (SPSS Inc., Chicago, IL, USA). Subsequently, we reviewed the cases of patients who showed hypokalemia during followup to assess the probable causes that affected the patients' potassium levels. When we reviewed the cases revealed hypokalemia, we used the Naranjo scale to estimate the influence of medication objectively.

## 3. Results

### 3.1. Patients' Characteristics

We identified 360 subjects fulfilling our criteria. The average dose of licorice was 8.7 ± 4.1 g/day (average ± standard deviation [SD]). Further, the average intake duration was 18.9 ± 19 days. The frequency of usage of herbal medicines was the highest for cerebral infarction (62.4%). Other characteristics of the patients at baseline are listed in Table 1.

### 3.2. Changes in the Levels of Potassium, BUN, and Creatinine

Although the potassium level decreased during follow-up examination, this decrease was not statistically significant (potassium levels before versus after, 4.00 ± 0.4 versus 3.99 ± 0.4; *P* = 0.815). BUN and creatinine levels also decreased. The levels of BUN showed a statistically significant decrease (before versus after, 13.91 ± 6.4 versus 12.64 ± 4.9; *P* < 0.001) (Table 2). However, all values were within the normal range (potassium 3.5~5.0 mEq/L; BUN 10~26 mg/dL; and creatinine 0.6~1.2 mg/dL).

### 3.3. Patients with Hypokalemia

Six patients (1.7%) had hypokalemia during the follow-up period (Table 3). The remaining 354 patients (98.3%) had normal levels of potassium during the intake of the herbal complexes.

The cases of patients with hypokalemia are as follows (Table 4).

Case 1 was a 78-year-old woman who presented dysarthria and right upper and lower extremities weakness with a history of cerebral infarction. Over a 22-day period, she received aspirin to prevent the recurrence of stroke and the herbal complex* Shipyukmiyouki-eum *to treat sequelae after cerebral infarction. During treatment with these agents, her potassium level decreased from 4.0 mEq/L to 2.5 mEq/L.

Case 2 was a 60-year-old woman diagnosed with cerebral hemorrhage, hypertension, and urinary tract infection. The patient was referred by the neurosurgery department to be treated with herbal complexes and acupuncture therapy to treat her left side paralysis and left shoulder pain caused by cerebral hemorrhage. She started treatment with antibiotics for the treatment of urinary tract infection that occurred during rehabilitation 5 days before administration of the herbal complex* Seogyung-tang*. Over a 6-day period, she received ciprofloxacin, ceftriaxone sodium, oxiracetam, and the herbal complex* Seogyung-tang*. During this series of treatments, her potassium level decreased from 3.5 mEq/L to 2.5 mEq/L.

Case 3 was a 56-year-old man diagnosed with cerebral hemorrhage and hypertension. The patient was admitted to treat hemiplegia and insomnia caused by cerebral hemorrhage. He had used lercanidipine HCl, atenolol, hydrochlorothiazide, and losartan potassium to control his hypertension and to prevent the recurrence of cerebral hemorrhage. After 14 days of onset of cerebral hemorrhage, he started treatment with* Gamiondamg-tang* to relieve insomnia over a 29-day period. During this treatment, his potassium level decreased from 3.9 mEq/L to 2.8 mEq/L.

Case 4 was a 77-year-old woman with cerebral infarction, hypertension, and diabetes. She was referred by the neurosurgery department for treatment of insomnia, palpitation, and hemiparesis caused by stroke. She had taken* S*-amlodipine, clopidogrel, calcium carbonate, pravastatin sodium, donepezil HCl, metformin HCl, and nicergoline to control the risk factors of stroke (hypertension, dyslipidemia, and diabetes mellitus). Over a 20-day period, she received the herbal complex* Gwibi-tang *to treat insomnia, palpitation, and sporadic diarrhea, which may be caused by metformin HCl. During this treatment, her potassium level decreased from 3.6 mEq/L to 2.6 mEq/L.

Case 5 was an 89-year-old woman with hypertension and coronary artery disease. After coronary angioplasty, she was referred by the cardiology department to treat the residual fatigue. At the time of admission, she had taken fluvastatin sodium, aspirin, nifedipine, atenolol, hydrochlorothiazide, tramadol HCl, acetaminophen, teprenone, and alfacalcidol. Over an 11-day period, she took the herbal complex* Bojungikki-tang* to treat her symptoms of fatigue. During this series of treatments, her potassium level decreased from 3.0 mEq/L to 2.2 mEq/L.

Case 6 was a 72-year-old woman diagnosed with cerebral infarction and hypertension, and she was referred by the neurosurgery department to treat sequelae of stroke after craniectomy. She had used quetiapine to treat delirium, gabapentin to treat neuralgia, aspirin to prevent stroke recurrence, and sodium valproate, dimethicone, hemicellulase, ox bile extract, and pancreatin. Over an 8-day period, she used the herbal complex* Banhasashim-tang *to treat her dyspepsia and abdominal discomfort. During this series of treatments, her potassium level decreased from 3.7 mEq/L to 2.8 mEq/L.

However, symptoms such as muscle weakness, hypertension, and renal failure were not observed in any of these 6 patients.

## 4. Discussion

In this study, we found no significant change in the potassium levels during the administration of herbal complexes containing licorice in 360 patients for 18.9 ± 19 days. Follow-up examination indicated that the potassium level decreased, but this decrease was not statistically significant (*P* = 0.815). The potassium levels were normal in 98.3% patients during the intake of herbal complexes. Only 1.7% patients had hypokalemia during followup. The average dose of licorice was 8.7 ± 4.1 g/day, and the average administration duration was 18.9 ± 19 days. Therefore, we suggested that administration of herbal complexes containing licorice could be safe when the dose of licorice was less than 8.7 ± 4.1 g/day and the duration of administration was less than 18.9 ± 19 days.

We observed 6 patients (1.7%) who developed hypokalemia after treatment with herbal medicine complexes containing licorice. However, we believed that the development of hypokalemia could not be attributed only to licorice in almost all patients (5 patients). We believe that concomitant administration of western medicines such as antihypertensive and antidiabetic agents and the medical condition of patients affected the development of hypokalemia.

Cases 3 and 5 took the antihypertensive agent hydrochlorothiazide. Diuretics such as thiazides are common cause of drug-induced hypokalemia [18]. Therefore, we assumed that long-term intake of hydrochlorothiazide can be a cause of hypokalemia in cases 3 and 5. Case 4 used metformin HCl to control her blood glucose levels. Before administration of the herbal medicine* Gwibi-tang*, the patient had sporadic diarrhea and soft stool. A previous case report indicated that a 57-year-old Caucasian male had hypokalemia, hypocalcemia, and hypomagnesemia induced by long-term administration of metformin HCl [19]. Therefore, we believe that long-term intake of metformin HCl can be attributed to the development of hypokalemia in case 4. Case 6 had used sodium valproate since craniectomy. A previous case report [20] indicated that a 14-year-old Japanese patient sometimes had fever and hypokalemia after intake of sodium valproate for 6 years. Therefore, we believe that consumption of sodium valproate can be a cause of hypokalemia in Case 6. In Case 2, fever caused by urinary tract infection may be attributed to the development of hypokalemia. Moreover, a previous study showed that the prevalence of hypokalemia in patients hospitalized for infection at an institution was 23% [21]. Thus, our results show that herbal complexes containing licorice cannot be a sole reason for the development of hypokalemia in these 5 patients.

Among the 360 patients, 1 patient (Case 1) had definite licorice-induced hypokalemia. In the case of this patient, no reasons other than the intake of* Shipyukmiyouki-eum* (licorice 8 g) could be attributed to the development of hypokalemia. This patient only used aspirin during* Shipyukmiyouki-eum* intake. No previous study to date suggests that aspirin may cause hypokalemia. Therefore,* Shipyukmiyouki-eum* can be a reason for hypokalemia.

Our study has the following limitations. We did not measure the serum levels of aldosterone, rennin, cortisol, and adrenocorticotropic hormone (ACTH). Therefore, we could not observe the influence of herbal complexes containing licorice on the endocrine system. Furthermore, because of retrospective nature of the study, we did not measure the amounts of glycyrrhetic acid which is the active component in licorice and duration of licorice contained herbal complexes intake and licorice dosage in each patients were not equal.

However, we believe that our findings can be a guide for safe use of licorice. We examined the average duration (18.9 ± 19 days) and dose of licorice (8.7 ± 4.1 g/day) use in these 360 patients. Our results revealed that severe hypokalemia did not occur during administration of herbal complexes containing licorice. Moreover, our study reflects the clinical circumstances better than those reported in previous studies. Doctors in Korea, Japan, and China who use herbal medicines to treat patients usually prescribe many types of herbs simultaneously. All patients in the present study consumed herbal complexes containing licorice. Previous studies have reported the cases of patients consuming only licorice [1, 4–17].

Our data suggest that administration of herbal complexes containing licorice does not affect the potassium levels when the dose of licorice is less than 8.7 ± 4.1 g/day and duration of administration is less than 18.9 ± 19 days. However, we think that hypokalemia in patients who use licorice can be made by interaction between licorice and drug which might cause electrolyte disturbances and renal function. Thus, we can assume that followup of potassium levels is required to prevent any unpredictable side effects of long-term administration of herbal complexes containing licorice in elderly people, especially, who used herbal medication with western medication.

Furthermore, we suggest that well designed prospective study, with standard administration (dosages of licorice and intake duration) and documented amount of glycyrrhetic acid, is necessary to solve the question of possible licorice induced side effects.

## 5. Conclusion

The present study assessed the influence of herbal complexes containing licorice on potassium levels. This study was conducted as a retrospective, chart review study. The results of this study are as follows.

1. Three hundred and sixty patients did not show significant changes in the levels of potassium and creatinine (*P* = 0.815, 0.289).
2. The average dose of licorice was 8.7 ± 4.1 g/day. Further, the average intake duration was 18.9 ± 19 days.
3. Six patients revealed hypokalemia. However, in 5 patients, the hypokalemia did not appear to be related to the herbal complex therapy containing licorice.

Therefore, we could suggest that administration of herbal complexes containing licorice does not affect the potassium levels at low dose and for a short period of time. However, we suggest that followup of potassium levels is needed to prevent any unpredictable side effects of administration of herbal complexes containing licorice.

## Figures and Tables

**Table 1:** Baseline characteristics (*n* = 360).

Characteristics	Frequency	Percentage %
Male/female	182/178	50.6/49.4
Age, years (Mean ± SD)	65.1 ± 13.9	
Causes of herbal medicine intake		
Cerebral infarction	224	62.4
Intracerebral hemorrhage	88	24.5
Brain tumor	2	0.6
Moyamoya disease	4	
Parkinson disease	4	1.1
Parkinsonism	5	1.4
Anoxic brain damage	23	6.4
Tension-type headache	1	0.3
Transverse myelitis	1	0.3
C-HNP	6	1.7
T-HNP	1	0.3
L-HNP	7	1.9
Spinal stenosis	4	1.1
Facial palsy	11	3.1
Lung cancer	3	0.8
Esophagus cancer	1	0.3
Colon cancer	1	0.3
Liver cancer	3	0.8
Kidney cancer	1	0.3
Gastric cancer	1	0.3
Gall bladder cancer	1	0.3
GERD	1	0.3
DVT	1	0.3
Osteoarthritis	3	0.8
CRPS	2	0.6
Licorice dose (per day) in g (Mean ± SD)	8.7 ± 4.1	
Duration of administration in days (Mean ± SD)	18.9 ± 19	

GERD: gastroesophageal reflux disease; DVT: deep vein thrombosis; CRPS: complex regional pain syndrome type; C-HNP: cervical herniated nucleus pulposus; T-HNP: thoracic cervical herniated nucleus pulposus; L-HNP: lumbar herniated nucleus pulposus.

**Table 2:** Measurement of the levels of potassium, blood urea nitrogen, and creatinine (*n* = 360).

Laboratory findings	First	Second	*P* value
Potassium level (mEq/L)	4.00 ± 0.4	3.99 ± 0.4	0.815
Blood urea nitrogen level (mg/dL)	13.91 ± 6.4	12.64 ± 4.9	<0.001
Creatinine level (mg/dL)	1.04 ± 7.4	0.6 ± 0.3	0.289

Values are average (standard deviation [SD]) and were compared using paired *t*-test.

**Table 3:** Incidence of hypokalemia (*n* = 360).

	Numbers (%)
Hypokalemia	6 (1.7)
Nonhypokalemia	354 (98.3)

Hypokalemia in this table indicates that potassium levels (at end point) are below 3.0 mEq/L.

**Table 4:** Occurrence of hypokalemia in patients after treatment with herbal complexes containing licorice.

Case number	Pot 1	Pot 2	Duration (days)	Causes of herbal medicine intake	Herbal complex taken, administration method (components in g/day)	Concomitant medications	Licorice doses per day	Estimate possibility/ Naranjo scale score
1	4.0	2.5	22	Cerebral infarction	*Shipyukmiyouki-eum*, oral administration (licorice 8 g, *Cinnamomum cassia *6 g, *Platycodi radix* 6 g, *Angelica gigas *12 g, *Aucklandia lappa* 6 g, *Saposhnikovia divaricata* 6 g, *Paeonia lactiflora* 6 g, *Angelica dahurica *6 g, *Areca catechu * 6 g, *Perilla frutescens* var. acuta 18 g, *Lindera aggregata* 6 g, *Panax ginseng* 12 g, *Citrus aurantium* 6 g, *Cnidium officinale* 6 g, *Astragalus membranaceus* 6 g, and *Magnolia obovata* 6 g)	Aspirin 100 mg	**8**	*Shipyukmi youki-eum*/2

2	3.5	2.5	6	Cerebral hemorrhage, hypertension, and **urinary tract infection**	*Seogyung-tang*, oral administration (licorice 6 g, *Ostericum koreanum *6 g, *Curcuma longa* 24 g, *Angelica gigas* 12 g, *Atractylodes japonica* 12 g, *Zingiber officinale* 12 g, *Paeonia lactiflora* 12 g, and *Kalopanax* species 12 g)	Ciprofloxacin 800 mg, ceftriaxone sodium 2 g, and oxiracetam 1600 mg	**6**	Urinary tract infection/—

3	3.9	2.8	29	Cerebral hemorrhage and hypertension	*Gamiondamg-tang*, oral administration (licorice 12 g, *Citrus unshiu* 15 g, *Platycodi radix* 8 g, *Liriope platyphylla* 8 g, *Pinellia ternata* 9 g, *Poria cocos* 8 g, *Bupleurum falcatum* 8 g, *Panax ginseng* 8 g, *Phyllostachys nigra* 9 g, *Citrus aurantium* 9 g, and *Cyperus rotundus* 9 g)	Lercanidipine HCl 10 mg, famotidine 40 mg, atenolol 25 mg, **hydrochlorothiazide** 12.5 mg, and losartan potassium 50 mg	**12**	Hydrochlorothiazide/2

4	3.6	2.6	20	Cerebral infarction, hypertension, and diabetes mellitus	*Gwibi-tang*, oral administration (licorice 6 g, *Angelica gigas* 6 g, *Aucklandia lappa* 12 g, *Poria cocos* 12 g, *Atractylodes japonica* 12 g, *Zizyphus jujuba* 12 g, *Dimocarpus longan* 12 g, *Polygala tenuifolia* 12 g, *Panax ginseng* 12 g, and *Astragalus membranaceus* 12 g)	S-Amlodipine 2.5 mg, clopidogrel 75 mg, Ca carbonate 500 mg, pravastatin sodium, 20 mg, donepezil HCl 5 mg, **metformin HCl,** 500 mg, and nicergoline 60 mg	**6**	Metformin HCl/2

5	3.0	2.2	11	Hypertension and coronary artery disease	*Bojungikki-tang*, oral administration (licorice 12 g, *Angelica gigas* 6 g, *Atractylodes japonica* 12 g, *Cimicifuga heracleifolia* 3 g, *Bupleurum falcatum * 3 g, *Panax ginseng* 12 g, *Citrus unshiu* 6 g, and *Astragalus membranaceus* 18 g)	Fluvastatin sodium 80 mg, aspirin 10 mg, nifedipine 33 mg, atenolol 25 mg, **hydrochlorothiazide** 12.5 mg, tramadol HCl 37.5 mg, acetaminophen 325 mg, teprenone 50 mg, and alfacalcidol 0.5 *μ*g	**12**	Hydrochlorothiazide/2

6	3.7	2.8	8	Cerebral infarction and hypertension	*Banhasashim-tang*, oral administration (licorice 12 g, Zingiberis Rhizoma Siccus 15 g, *Zizyphus jujuba *12 g, *Pinellia ternata *30 g, *Panax ginseng* 15 g, *Scutellaria baicalensis* 15 g, *Coptis japonica *6 g)	Quetiapine 25 mg, Gabapentin 900 mg, aspirin 100 mg, **sodiumvalproate** 1800 mg, dimethicone 75 mg, hemicellulase 150 mg, ox bile extract 75 mg, and pancreatin 525 mg	**12**	Sodium valproate/2
